# A multi-colour fluorogenic tag and its application in Candida albicans

**DOI:** 10.1099/mic.0.001451

**Published:** 2024-03-27

**Authors:** Jonas Devos, Patrick Van Dijck, Wouter Van Genechten

**Affiliations:** 1 Laboratory of Molecular Cell Biology, Institute of Botany and Microbiology, KU Leuven, 3001 Leuven, Belgium

**Keywords:** *Candida albicans*, fluorogenic tag, iFAST, microscopy

## Abstract

Fluorescent proteins (FPs) have always been a crucial part of molecular research in life sciences, including the research into the human fungal pathogen *Candida albicans,* but have obvious shortcomings such as their relatively large size and long maturation time. However, the next generation of FPs overcome these issues and rely on the binding of a fluorogen for the protein to become fluorescently active. This generation of FPs includes the improved version of Fluorescence activating and Absorption Shifting Tag (iFAST). The binding between the fluorogen and the iFAST protein is reversible, thus resulting in reversible fluorescence. The fluorogens of iFAST are analogues of 4-hydroxylbenzylidene-rhodanine (HBR). These HBR analogues differ in spectral properties depending on functional group substitutions, which gives the iFAST system flexibility in terms of absorbance and emission maxima. In this work we describe and illustrate the application of iFAST as a protein tag and its reversible multi-colour characteristics in *C. albicans*.

**Impact Statement**

*Candida albicans* is a commensal of the human host with the ability to become pathogenic, which can result in systemic disease with high mortality. A substantial amount of research has already been done in regard to its virulence factors and underlying molecular mechanisms, but a lot remains unclear. An important aspect of current molecular research is fluorescence microscopy and fluorescent probes. Due to *C. albicans* being long overlooked as a significant pathogen, and because of its aberrant codon usage, the palette of optimised fluorescent proteins and biosensors for this organism is quite limited compared to the bacterial palette or the tags available for model organisms like *Saccharomyces cerevisiae*. In this work we present the use of a novel multi-colour fluorescent protein, which has previously been utilized in both living and fixed cells. Use of this novel protein allows researchers to tailor a combination of iFAST and fluorogen that optimally fits their purpose in multi-colour imaging of *C. albicans*.

## Data Summary

The authors confirm all supporting data, code and protocols have been provided within the article or through supplementary data files.

## Introduction

*Candida albicans*, a common commensal found in the gastrointestinal tract, has the ability to cause benign skin infections as well as severe infections in the vaginal and oral mucosa [1,3]. While the immune system of healthy individuals can efficiently handle these fungal infections, *C. albicans* can cause invasive infections with a high mortality rate under opportunistic circumstances. Factors that contribute to this fungal susceptibility include immunocompromising diseases like HIV/AIDS, disruption of the microbiome, treatment with immunosuppressants, or invasive medical procedures [4].

Despite the significant morbidity associated with fungal infections, opportunistic fungal pathogens such as *C. albicans* have historically received less attention, resulting in a research gap compared to studies on mammals or bacteria. This lack of focus is also evident in the limited availability of optimised molecular tools, including fluorescent proteins (FPs), specifically tailored for *C. albicans* when compared to the model eukaryote *Saccharomyces cerevisiae* [5,8]. Furthermore, the optimization of these tools for *C. albicans* is hindered by the fact that *C. albicans* translates the CUG codon to serine instead of leucine in 96 % of the cases [9,10]. As a result of this alternative codon usage, *C. albicans* genes and proteins cannot easily be studied in model organisms such as *S. cerevisiae* as these will be mistranslated. The alternative usage of the CUG codon has an impact on the usability of a lot of molecular and fluorescent tools, as these are most often developed for use in mammalian or bacterial cells and will therefore suffer from the same mistranslation. Several techniques for codon optimization have been reported [11,12], but these have been shown to not be generally applicable for *C. albicans* [13].

One of the major challenges in the field of *C. albicans* research is the discovery of novel anti-fungal targets [14]. A popular way of doing this is by making use of molecular tools like FPs to study the localization and expression of potential targets and interesting pathways. In recent years a novel class of FPs, based on fluorogens, has emerged [6,15, 16]. These FPs differ from the classical GFP-type FPs in that they require the binding of their fluorogen to become fluorescently active. An example of such an FP is Fluorescence activating and Absorption Shifting Tag (FAST) [6]. Fluorescence of this FP is achieved by the binding of 4-hydroxylbenzylidene-rhodanine (HBR) analogues into the pocket of the FAST protein. Different HBR analogues are available, but this work focuses on the use of 4-hydroxy-3-methylbenzylidene-rhodanine (HMBR, further referred to as Lime), 4-hydroxy-3,5-dimethylbenzylidene-rhodanine (HBR-3,5DM, further referred to as Amber) and 4-hydroxy-3,5-dimethoxylbenzylidene-rhodanine (HBR-3,5DOM, further referred to as Coral). The major advantages of this protein tag are its small size and its rapid, reversible fluorescent signal. The reversibility dynamics of the system are dependent on the fluorogen, the FAST:HBR combination has a relaxation time of binding (when [HBR]=K_D_) and residence time of 30 ms and 60 ms respectively at 25 °C. However, FAST has a higher affinity for Lime, which increases its relaxation and residence times to 60 ms and 160 ms respectively at 25 °C [6]. After the initial development of FAST, several different versions have been reported, including an improved version with brighter fluorescence (iFAST). Using rational design iFAST was derived from FAST by a single amino acid mutation (V107I) [5]. Although the focus of this report is on live-cell imaging, FAST has been used in imaging of fixed cells, showing its potential for single molecule localization microscopy (SMLM) [17].

To further expand and advance the palette of optimised fluorescent tools for *C. albicans* we optimised iFAST and characterized its reversibility and stability in *C. albicans*. Additionally, we exemplified its use as a protein tag in *C. albicans* by tagging lanosterol 14α-demethylase (Erg11), the main target of the azole class of antifungals, and Ftr1, which is an iron permease shown to contribute to the virulence of the fungus [18].

## Methods

### Construction of cytosolic iFAST expressing strains

The codon optimised FAST sequence for use in *C. albicans* was derived from the *S. cerevisiae* optimised sequence by replacing all CUG codons for UUA [6]. Thereafter, the iFAST sequence was obtained by exchanging a GTC codon to ATT codon resulting in the V107I mutation (Supplementary information 1). This sequence for iFAST was amplified from a gBlock (IDT) via PCR to have flanking sequences allowing for Gibson assembly into the fluorophore cloning site, placing it under the control of the *MET*3p, of the 2015-N plasmid for cytosolic expression (for primers see Table S1, available in the online version of this article) [19]. Before insertion of the iFAST sequence, the 2015-N plasmid (Table S2) was digested using BspeI and NheI, a step that was omitted for the empty vector strain. In case of the 2015N-Venus plasmid the iFAST sequence was replaced with a *C. albicans* optimized version of Venus. To transform these vectors into the SN152 strain (Table S3), an optimised heat shock protocol of Gietz and colleagues was followed [20,21]. Correct strains were selected based on growth on solid medium lacking leucine and a PCR (Fig. S1A) with diagnostic primers listed in (Table S1).

### Construction of endogenously tagged *C. albicans* strains

Endogenous tagging of *FTR1* was done through transformation of a linear cassette amplified from a pFA6-based plasmid containing the iFAST sequence. This plasmid was constructed through amplification of an iFAST gBlock for Gibson assembly (using primers listed in Table S1), before insertion into a linearised pFA6 plasmid (Table S2) which was digested using PstI-HF and AscI. A linear cassette was constructed through PCR on pFA6-iFAST to have a 50 bp overlap with the end of Ftr1, excluding the stop codon, and the start of its terminator. The forward primer contained a double linker, consisting of four glycines and one serine, between the end of Ftr1 and the beginning of iFAST. These linear cassettes were integrated into the genome of the SC5314 wild-type strain using the optimised Gietz transformation protocol. For endogenous tagging of Ftr1 with mTurquoise2 an identical approach was utilized with a pFA6 – mTurquoise2 plasmid reported by our laboratory [13]. Strains were selected on nourseothricin containing media and subjected to diagnostic PCR (Fig. S1B).

### Generation of tagged overexpression strains

The *ERG11*-iFAST and *ERG11*-GFP overexpression constructs were generated by amplifying *ERG11* and respectively iFAST or GFP with flanking sequences for Gibson assembly from WT genomic DNA and a gBlock respectively (primers listed in Table S1). The overexpression plasmid was generated by insertion of both PCR products into a CIp10 plasmid, where the *URA3* marker was replaced with a *NAT1* gene conferring resistance to nourseothricin, digested with PstI and NheI, using the NEBuilder HiFi DNA assembly kit. For transformation into *C. albicans* WT strain SC5314, the plasmid was linearised using StuI. Following transformation, selection was performed on solid medium with nourseothricin and diagnostic PCR (Fig. S1C).

### Strains and growth conditions

The plasmids constructed during this work were transformed and integrated into the SN152 or the SC5314 strain. For visualization of the tagged protein constructs and the growth assays, strains were grown overnight in low fluorescence medium containing 0.69 % (w/v) Yeast Nitrogen Base without amino acids, ammonium sulphate and riboflavin, 0.079 % (w/v) complete supplement mixture (CSM) powder, 0.5 % (w/v) ammonium sulphate and 2 % (w/v) glucose. For the reversibility experiments the overnight culture was grown in complete medium lacking methionine and cysteine. This drop-out medium consisted of 0.17 % (w/v) Yeast Nitrogen Base without amino acids and ammonium sulphate, 0.183 % (w/v) CSM-Met-Cys, 0.5 % (w/v) ammonium sulphate and 2 % (w/v) glucose.

### Confocal microscopy

Where cells were required for confocal microscopy, the cell culture was grown overnight in liquid low fluorescence medium and diluted to an optical density (OD) at 600 nm (OD_600_) of 0.2 in fresh low fluorescence medium and grown to the mid-exponential phase (4–5 h). After an incubation period of 15 min, unless stated otherwise, with one of the fluorogens, the strains were imaged on glass slides using an Olympus Fluoview FV1000 inverted epi-fluorescence microscope equipped UPLSAPO 60X objective (NA=1.34). A 488 nm laser used for the Lime or Amber fluorogens, and a 515 nm laser when using the Coral fluorogen, both lasers emit a theoretical total of 100 µW from the objective. The 488 nm laser was utilised in combination with a 505–565 nm bandpass filter for the emitted light, whilst the 515 nm laser was utilised in combination with a 575–620 nm bandpass filter both set at a detector high voltage (hv) setting of 580 for the photomultiplier tube (PMT). All images taken were the result of a single focal plane.

### Localization assay

Localization of iFAST tagged proteins was assessed by comparing an iFAST tagged strain (either Erg11 or Ftr1) to a non-tagged control (SC5314). Fluorescence images were acquired via confocal microscopy from cells cultured in low fluorescence medium containing 50 µM of fluorogen (Lime or Coral in the case of Ftr1 – iFAST and Erg11 – iFAST, respectively). From each strain a minimum of 37 cells were analysed, obtained from multiple fields of view of which representative images and complete data analysis is reported.

### Reversibility of the fluorescence signal

To examine the contrast, staining efficiency and reversibility of the iFAST system in *C. albicans*, both WT and an iFAST integrated strain were subjected to the fluorogens at 50 µM for a period of 15 min. The iFAST integrated strain, utilized in all experiments to assess reversibility, expresses cytosolic iFAST in medium lacking methionine and cysteine. At predefined timepoints (2, 5, 10 and 15 min) cells were isolated from the culture and imaged. The strains were subsequently subjected to two wash steps, and three images were taken 5 min after each of the wash steps. Furthermore, to examine whether the reversibility of FAST allows for removal of the fluorogen and subsequent addition of another fluorogen, we performed an experiment according to the following scheme: Lime is added, then washed from the cells, subsequently Coral is added before another round of washing to finally administer Lime again. Finally, to assess whether washing results in a significant drop in fluorescence compared to a non-washed condition we performed a long-term experiment where we compared cells from a Lime- or Coral-administered culture with a double-washed culture at different time points.

From each timepoint listed in the experimental setup above, the cells of the images were pooled, resulting in an average of 45 analysed cells per strain per timepoint. As a reference point the fluorescence of each strain was assessed at the beginning of the experiment before addition of the fluorogen. Fluorescence images were acquired via confocal microscopy. To calculate the fold change in fluorescence overtime, the fluorescence intensities of all cells were calculated for each image and normalized to the mean fluorescence of the same strain before the beginning of the experiment, indicated as timepoint -5. The upper and lower limits of the display range of all images were set into accordance with the minimum and maximum intensity of the microscope (0 and 4095 respectively). The fluorescence intensity was assessed by calculating the mean grey value of all cells individually using manually selected regions of interest (ROI) in the DIC channel. The ROIs were transferred to the adjusted 16-bit grey scale fluorescence channel and used to calculate the mean intensity of all cells individually. To account for variation in background noise, the average of seven ROIs, which are similar in size to the yeast cells, of the background was subtracted from all individual cell measurements. We omitted cells from the analysis that were out of focus or showed aberrant morphology. All image processing and fluorescence measurements were performed using ImageJ2 (v2.14.0/1.54 f) [22].

### Growth assay

To assess the effect of the fluorogens on the growth of *C. albicans*, the SN152 strain with integration of the iFAST sequence, was grown in low fluorescence medium with one of the fluorogens in different concentrations (0, 10, 20, and 50 µM, dissolved in DMSO). Overnight growth conditions are similar to those stated earlier. Before the start of the experiment the strain was diluted to OD_600_ of 0.01 in fresh low fluorescence medium with different concentrations of fluorogen (at 0, 10, 20 or 50 µM). In case of the control condition with 0 µM of fluorogen, the same volume of DMSO as in the 50 µM condition was added to the medium. The growth was monitored in a flat bottom 96-well plate by measuring the absorbance at 595 nm over a period of 3 days using a Multiskan FC. In addition, a growth assay was performed to compare the growth of the iFAST-expressing strain to the wild-type, an empty vector (EV) control and a Venus-expressing strain, in low fluorescence medium lacking methionine and cysteine. All growth assays have three biological repeats with a minimum of two technical repeats. Additionally, three blanks of each condition, not containing any *C. albicans* cells, were used to subtract any background absorbance coming from either the medium or the fluorogen. After calculating the average of all technical repeats, the final absorbance of the biological repeats was calculated by subtracting the mean absorbance of the blank conditions. As an approximation of the growth rate, the slope of the linear phase of the growth curve was calculated. Using the R package ‘QurvE’ (v1.1) the slope of the linear phase and the lag times of each strain was calculated and statistically compared via a one-way ANOVA with a Tukey post-hoc test.

## Results

### iFAST fluorogens have a slight impact on *C. albicans* growth

A prerequisite of a good molecular tool is one that does not heavily interferes with the growth or molecular processes of the cell. To monitor potential adverse effects of the fluorogens on the growth of *C. albicans*, three biological repeats, each with two technical repeats, of the iFAST integrated strain were grown in different concentrations of the three fluorogens (Lime, Amber or Coral). To ensure that only the effect of the fluorogens on the growth was measured, the cells were grown in low fluorescence medium supplemented with all needed amino acids. Furthermore, we also investigated whether the vector integration and expression of iFAST, in the absence of the fluorogens, had any adverse effects on the growth.

**Fig. 1. F1:**
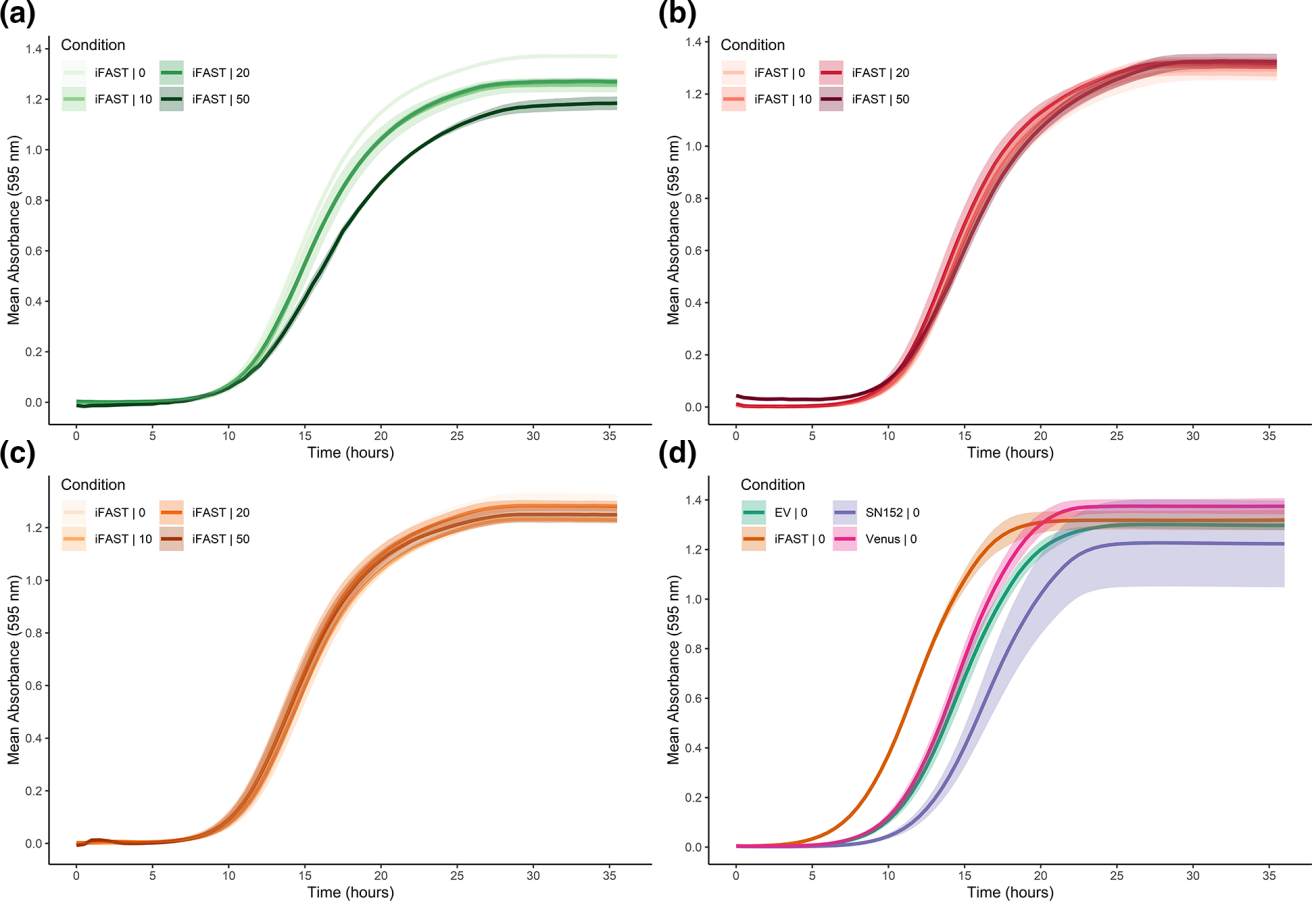
Assessment of iFAST fluorogen toxicity on *C. albicans*. Growth curves of iFAST grown in different concentrations (0, 10, 20 or 50 µM) of Lime (**a**), Coral (**b**), and Amber (**c**). The iFAST strain was grown in low fluorescence medium overnight and diluted the next morning to an OD_600_ of 0.01 in fresh low fluorescence medium. The growth at 30 °C was monitored by measuring the absorbance at 595 nm every 30 min. The presented data shows the mean and standard deviation of three biological repeats with two technical repeats and the standard deviation between the biological repeats. Data analysis was done via a one-way ANOVA test with Tukey post-hoc testing (Table S4). (**d**): Growth comparison of the iFAST integrated strain with the SN152 background, and empty vector (EV) control and a Venus integrated strain. Growth conditions are similar to a, b and c, except medium lacking methionine and cysteine was used. The data shows the mean and the standard deviation from two independent experiments with three biological repeats and each a minimum of two technical repeats. Data analysis was done via a one-way ANOVA test with Tukey post-hoc testing (Table S4).

For the analysis of the fluorogen effect on growth, the data was grouped per fluorogen and compared (Fig. 1a–c).Fi[Fig F1]g. 1a[Fig F2]-c For all fluorogens similar growth patterns were observed. However, from the figures we can already observe that at higher concentrations of fluorogen the growth curves are somewhat distinct, and this is most apparent in the case of Lime (Fig. 1a). Data analysis indicated that 50 µM of Lime and Coral significantly reduced the growth rate compared to the 0 µM control (*P*=0.0104 and *P*<0.0001). For iFAST with Amber none of the fluorogen concentrations had any significant effect on growth rate compared to the 0 µM condition.

To rule out any adverse effects of vector integration and iFAST expression, the growth of the iFAST integrated strain was compared to the wild-type and the empty vector control (EV). Additionally, a strain integrated with *C. albicans* optimized Venus was taken along (Fig. 1d). The comparison showed that the reintroduction of one of the auxotrophic markers, which is the case for the iFAST, the Venus integrated strain, and the EV strain has a positive effect on both the growth rate and the lag phase. While data analysis indicated an overall significant effect of the strain on the growth rate (*P*=0.042), no significant differences in growth rate were found to the wild-type strain. However, the lag phase was significantly shorter for each of the vector integrated strains compared to the wild-type (iFAST vs SN152: *P*<0.0001, EV vs SN152: *P*=0.0124, Venus vs SN152: *P*=0.0047). Most importantly, expression of iFAST did not result in a significant difference in growth rate compared to the empty vector control (*P*=0.0609) and even resulted in a significantly shorter lag phase (*P*<0.0001). The reason behind this shorter lag phase remains unclear.

### iFAST fluorescence is reversible

**Fig. 2. F2:**
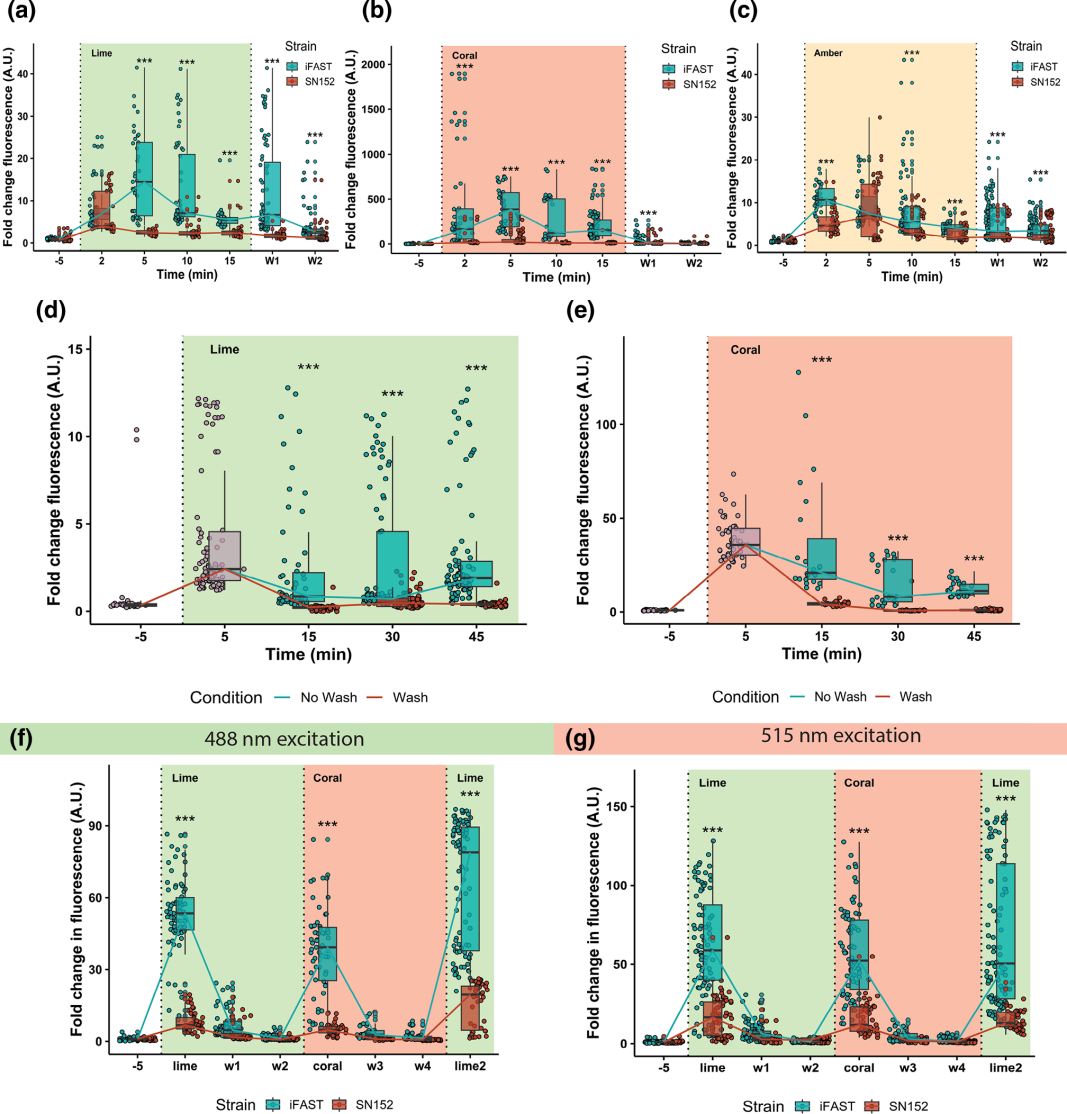
Reversible fluorescence of iFAST by addition and removal of the fluorogen. SN152 and the iFAST integrated strain were grown overnight at 30 °C in medium lacking methionine and cysteine in order to cytosolically express iFAST. Cells were diluted to an OD_600_ of 0.2 before growing to mid-exponential phase. Of each strain the fluorescence was measured before, during and after addition of the fluorogen. At minute 0, the strains were subjected to 50 µM of fluorogen (Lime, Coral, and Amber; respectively in panel A, B, and C). After 15 min the strains were subjected to two wash steps, after each of which the fluorescence was assessed. In panels D and E respectively, cells were subjected to Lime or Coral for 45 min and compared with cells washed after 5 min. Panels F and G depict a reversibility experiment where cells were initially subjected to Lime, washed twice, subjected to Coral, washed twice, before finally being subjected to Lime. Data in panel F is measured using settings appropriate for Lime. Data in panel G is measured with settings appropriate for Coral. The box plots and the connecting lines correspond to the median fold change in fluorescence and each dot represents a single cell. Statistical testing for panels A-G consisted of t-tests to assess the differences in fluorescence between the iFAST and SN152 strain during each fluorogen treatment or timepoint. Asterisks indicate significance levels; ≤ 0.5: *; ≤ 0.1: **; ≤ 0.001: ***. For panel A, B, C, D, E, F and G, on average 45 cells were analysed for a strain at each certain timepoint and each individual cell is represented as a dot within each boxplot.

What makes iFAST stand out as a fluorescent labelling technique is its reported reversibility. This characteristic gives the system a high tunability both in spectral properties and temporal control of the fluorescence. To assess this reversibility in *C. albicans*, a WT and an iFAST integrated strain were subjected to 50 µM of each of the fluorogens and fluorescence was assessed at 2, 5, 10 and 15 min before the cells were washed. After calculation of the mean fluorence intensity (MFI) of a minimum of 57 cells per time point, the fold change in fluorescence of each strain was plotted per fluorogen (Fig. 2a–c).

On the graphs of Lime and Coral a similar pattern in fold change fluorescence can be observed. This pattern consists of a rapid increase in fluorescence followed by a steady decline, the decline is extended during the two wash steps. In the case of Lime (Fig. 2a), a significant difference is observed between the iFAST strain and the untagged parent strain at 2, 5, 10 and 15 min. After a first wash step (W1) there is still a significant difference between the iFAST and SN152 (*P*<0.0001), indicating that in this experiment a single wash was insufficient. Even after a second wash step there is still an amount of Lime dye remaining in the iFAST tagged strain, resulting in a significant difference in fluorescence obtained from the iFAST tagged strain compared to the SN152 (*P*=0.0002). For Coral (Fig. 2b) a similar pattern is observed, but the significant difference between the tagged and non-tagged strain is eliminated after two washes. For the cells imaged with Amber (Fig. 2c) the trend is not as clear even though significant differences are observed between the iFAST and SN152 strains. The SN152 treated strain accumulates some fluorogen leading to an approximate five- to 15-fold increase in fluorescence compared to the initial non-Amber condition. This accumulation of Amber in the SN152 strain is also apparent in (Fig. S2). For this reason, Amber will not be utilized in the remainder of this study. The level of fluorescence and the speed with which maximum fluorescence is achieved after administration of fluorogen differs between the different strains and the different fluorogens, with the iFAST integrated strain with Coral achieving a median 380-fold change in fluorescence after 5 min (Fig. 2b). This fold change in fluorescence is the largest contrast observed between the WT and the iFAST integrated strain. Additionally, 18.6 % of cells from the integrated strain were found to have a fold change larger than 1000 after 2 min.

Since some decay in fluorescence is observed over time in the iFAST tagged strains we performed an experiment where we compare iFAST-expressing cells that are continuously exposed to the HBR analogues with those that were exposed to the fluorogen for 5 min and then washed twice. In (Fig. 2d) we can see that the iFAST expressing cells show an initial increase in fluorescence upon administration of Lime and then similarly to (Fig. 2a) display a drop in fluorescence. However, the level of fluorescence remains relatively stable over the 15-to-45-minute period. Furthermore, there is a significant difference between the ‘No Wash’ and ‘Wash’ cells at the 15, 30 and 45 min markers (*P*<0.0001 at each timepoint). In (Fig. 2e) a similar trend is observed when we do a similar experiment but with Coral as the fluorogen. A significant difference is observed between the ‘No Wash’ and ‘Wash’ cells at the 15, 30 and 45 min markers (for all three *P*<0.0001).

To further characterize the multi-colour aspect of the iFAST tag we performed sequential staining of the cytosolically expressing iFAST strain using a Lime – Coral – Lime scheme with two wash steps in between each fluorogen. We performed this experiment with microscope settings most suitable for Lime and Coral, respectively as shown in panel F and G. From panel F we can observe that addition of Lime results in a sharper significant increase in fluorescence of the iFAST expressing strain compared to the SN152 control (*P*<0.0001) which can be washed out by two sequential washes that drops the fluorescence signal. After this decline in fluorescence due to washing, addition of Coral leads to an increase in fluorescence which is stronger in the iFAST expressing strain compared to the SN152 (*P*<0.0001). This increase in fluorescence is measured using settings for Lime imaging (488 nm excitation, 505–565 emission filter). This suggests that there is relatively large bleed through from the Coral fluorogen into the Lime channel. Again, washing steps result in a decrease of fluorescence and addition of Lime results in a large significant fluorescence difference between the iFAST and SN152 strain (*P*<0.0001).

In (Fig. 2) panel G the results from the same experiment are depicted but measuring fluorescence with settings suitable for Coral (515 nm excitation, 575–620 emission filter). Similar trends to panel G are observed with significant differences for the iFAST and SN152 strain after initial administration of Lime (*P*<0.0001), after administration of Coral after the second wash step (*P*<0.0001) or after the fourth wash step (*P*<0.0001). Bleed through from the Lime fluorogen into the Coral channel is also observed.

### Protein localization using iFAST

**Fig. 3.**
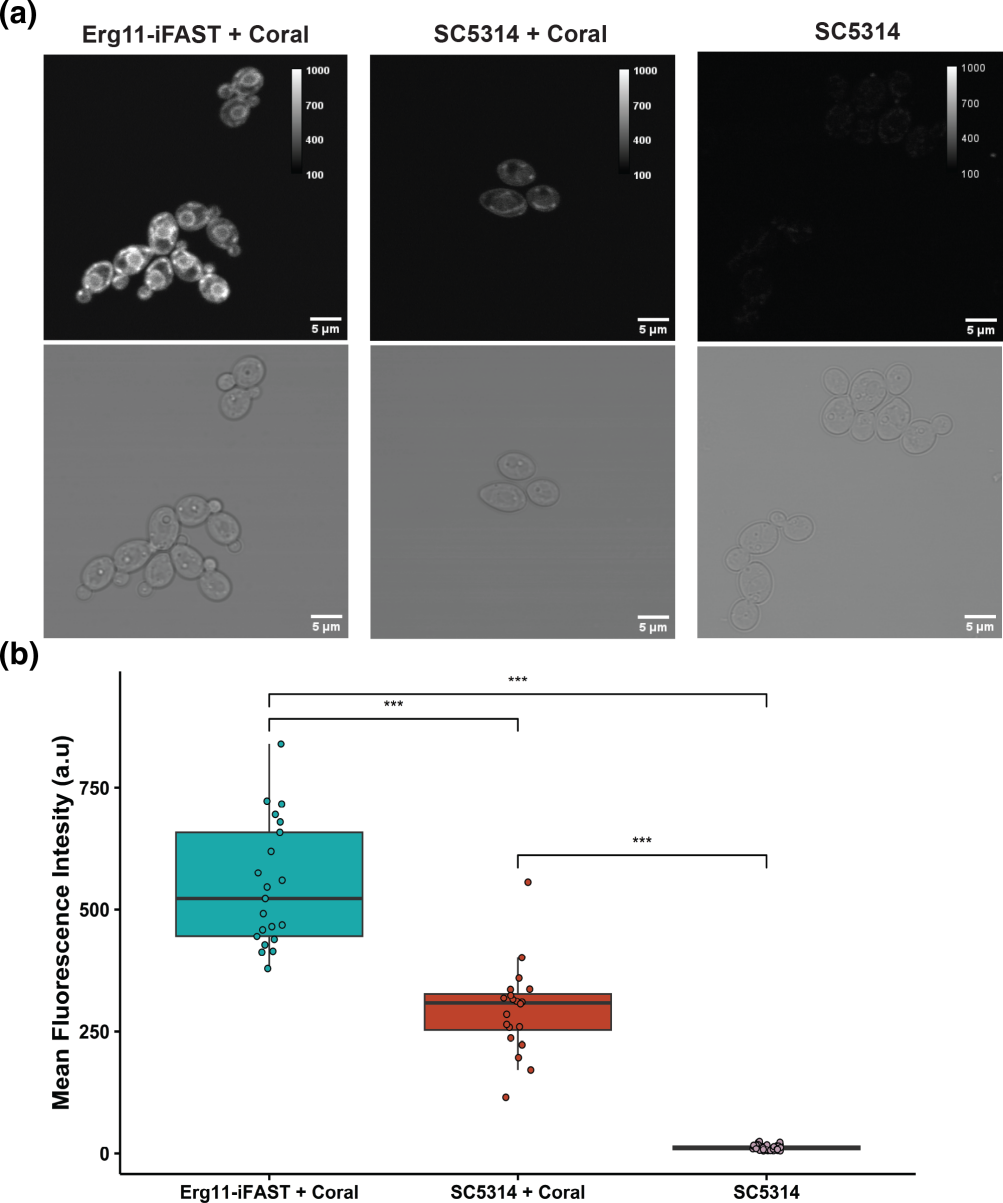
Overexpression strain of Erg11 tagged with iFAST:Coral. SC5314 with and without integration of the ERG11-iFAST plasmid were grown overnight at 30 °C in low fluorescence medium to mid exponential phase before imaging. Confocal images were acquired from a single-slice after 15 min of incubation with 50 µM Coral fluorogen. (**a**) Representative images of Erg11-iFAST strain with Coral (left), SC5314 with Coral (middle) and SC5314 without Coral (right). (**b**) quantification of a series of images obtained with the strains depicted in panel (a). The centre of the box plots correspond to the median fluorescence intensity. Significance was calculated using an ANOVA with a Tukey post-hoc test. Stars denote signficance; *P*≤0.5: *; ≤ 0.1: **; ≤ 0.001: ***. At least 20 cells from three biological repeats were measured for each condition.

When using a FP to tag an endogenous protein it is important that the FP does not interfere with the functionality, folding and localization of the target protein. As iFAST is a smaller protein, there is less chance of this protein to hinder the natural function or localization of the tagged protein [6]. To assess the applicability of iFAST as a protein tag in *C. albicans*, Erg11 (Erg11-iFAST) and Ftr1 (Ftr1-iFAST) were tagged, using overexpression and endogenous constructs respectively. Both constructs contain a *SAT1* gene conferring nourseothricin resistance, based on which selection was done after transformation and integration. Successfully transformed strains were subsequently checked with diagnostic PCR (Fig. S1) and imaged with fluorescence confocal microscopy. To test the flexibility of the iFAST system, Ftr1-iFAST and Erg11-iFAST were imaged with Lime and Coral respectively. From each strain in (Figs3  4) a minimum of 20 cells were analysed over three biological repeats, the cells shown are representative of all analysed cells.

**Fig. 4.**
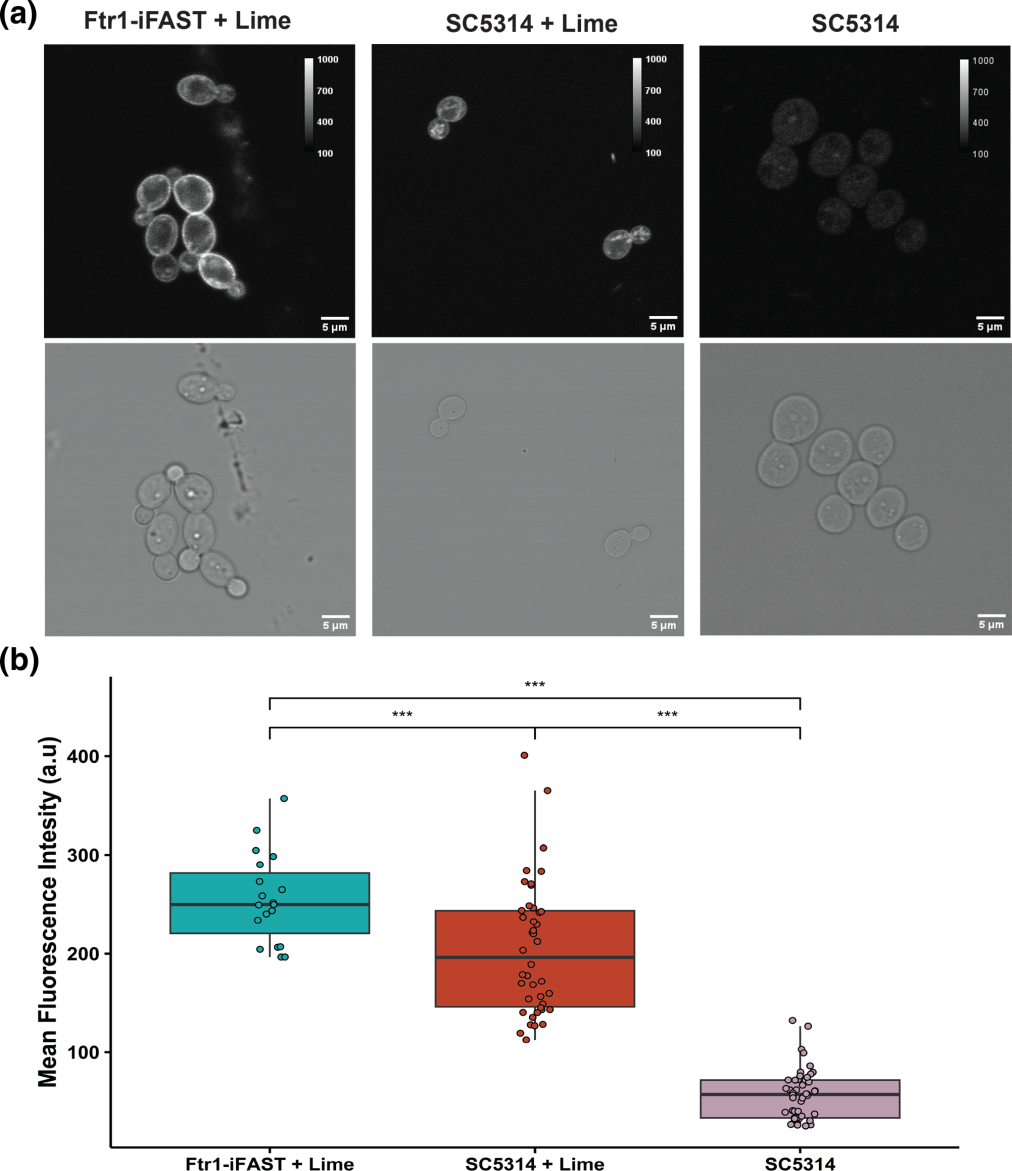
Endogenous tagging of Ftr1 using iFAST:Lime. Strains were grown overnight at 30 °C in low fluorescence medium to mid exponential phase before addition of the fluorogen and imaging. Confocal images were acquired from a single-slice after 15 min of incubation with 50 µM Lime fluorogen. (**a**) Representative images of Ftr1-iFAST strain with Lime (left), SC5314 with Lime (middle) and SC5314 without Lime (right). (**b**) quantification of a series of images obtained with the strains depicted in panel (a). The centre of the box plots correspond to the median fluorescence intensity. Significance was calculated using an ANOVA with a Tukey post-hoc test. Stars denote signficance; *P*≤0.5: *; ≤ 0.1: **; ≤ 0.001: ***. At least 20 cells from three biological repeats were measured for each condition.

As expected, in the Erg11-iFAST strain we found localization of fluorescence at the ER and cortical ER (Fig. 3a) [23,24]. A significant difference is observed in mean fluorescence acquired from the Erg11-iFAST strain compared to the parent SC5314 treated with Coral (*P*<0.0001). A significant difference in fluorescence is also observed in the SC5314 treated with or without Coral (*P*<0.0001), indicating that addition of Coral increases the background signal in SC5314, which is visible in (Fig. 3a).

In the case of the Ftr1-iFAST strain, the signal was found to be localised mainly at the plasma membrane (Fig. 4a) [18]. From the quantification of the fluorescence signal depicted in (Fig. 4b) we can observe some background signal in the SC5314 strain which has been stained with Lime, however a significantly higher signal is obtained from the Ftr1-iFast strains (*P*=0.00078). The background signal observed in the SC5314 +Lime condition is significantly higher than the unstained SC5314 (*P*<0.0001). It is apparent in (Fig. 4b) that the SC5314 strain stained with Lime does show a wide variability in fluorescence intensity with some higher intensity cells. For clarity some of these higher intensity cells are shown in panel A (middle). For both fusion constructs the fluorescence pattern was clearly distinguishable from the intracellular background fluorescence of the WT control, where only some intracellular fluorescence was found closely resembling mitochondrial strands (Figs3b  4b).

In addition to fluorescence localization, growth of the two strains was compared to the growth of to the wild-type and the growth of a strain tagged with an alternative FP (GFP and mTurquoise2 in case of Erg11 and Ftr1 respectively). Results are shown in (Fig. S3). Since in the case of the Erg11 overexpression constructs a linearized complete plasmid is integrated into the *RPS10* locus, an additional empty vector control was used. Statistical analysis on the data showed no significant differences in growth rate for Ftr1 strains (Table S4). However, the strain overexpressing Erg11-iFAST showed a reduced growth rate compared to the control conditions (Erg11-iFAST vs EV; *P*=0.0037, Erg11-iFAST vs Erg11-GFP; *P*=0.0067).

## Discussion

Fluorescent proteins and fluorescence microscopy are a standard practice for molecular cell biology research to acquire novel insight into molecular mechanisms. Research on the human fungal pathogen, *C. albicans*, has greatly benefited from fluorescence microscopy to elucidate the regulation of virulence factors [25,27]. To further advance this research field it is important to have a broad palette of options, both in fluorescent proteins and probes as well as molecular tools. In this paper we demonstrated the use of iFAST as a novel, small, reversible fluorescent protein tag in *C. albicans* yeast cells. Hyphal cells and the influence of fluorogens on hyphal growth and extension rate are outside the scope of this report and requires a thorough study in itself.

As stated above, we want minimal influence of the iFAST system on *C. albicans*. From the growth assay we only saw a significant reduction in growth rate, compared to the other conditions, by addition of 50 µM of Lime and Coral to the iFAST integrated strain. These results indicate a long-term effect on growth of the fluorogens at higher concentrations on *C. albicans*. However, during our experiments the strains are incubated with the fluorogens for no longer than 45 min, therefore we do not expect any immediate severe adverse effects. Nevertheless, we cannot elucidate and therefore deny the effect of the fluorogen on all the attributes composing total cell fitness, but these effects could also be the case for any genetic manipulation or tag. When it comes to the integration of iFAST into the SN152 strain, no significant reduction is seen in growth rate. When it comes to the lag phase, the strains containing an integration with a 2015 plasmid had a reduced lag time, which is most likely due to the reintegration of the *LEU2* gene. At this point however, we do not have an explanation for the observed shorter lag phase of the iFAST-expressing strain compared to the Venus-expressing strain or the empty vector.

By measuring fluorescence before, during and after addition of the fluorogens, we showed that the iFAST system in *C. albicans* is reversible, although not always completely recovering to fluorescence levels resembling non-stained conditions, and that this system can produce high contrast compared to the non-tagged control condition. Even though we saw high fluorescence gains upon addition of fluorogen to iFAST expressing strains (Fig. 2), we observed some variation in the level of fluorescence between cells as can be seen from the boxplots, whiskers and outliers. Furthermore, a decline in fluorescence independent of the wash step was observed after 10 min. Strikingly, this decline in fluorescence over time has not been reported before for any of the FAST versions when used in mammalian cells [5,6, 28, 29]. To assess if this decrease in fluorescence still results in sufficient contrast with washed cells, we compared fluorogen-subjected cells with fluorogen-treated and consecutively washed cells. The washed cells are significantly dimmer compared to non-washed cells (Fig. 2d, e), indicating that washing does result in reversibility of the fluorogen:iFAST binding and provides sufficient contrast. Within this washed to non-washed comparison we also assessed longer term stability of the signal. We can see that after the initial increase in fluorescence, the signal drops but remains relatively stable throughout the 15-to-45-minute period. This indicates that imaging the cells within 5 min may result in the highest signal, but some of this may be non-specific. Another possibility is that *C. albicans* may actively remove some of the fluorogen via drug efflux pumps. Depending on the research question at hand and/or practical concerns, imaging seems to be possible at all time points as illustrated in this report.

Although the original publications on FAST and iFAST show promising results, some extent of fluorescence variation is also visible in the reported images when versions of FAST are expressed cytosolically in mammalian cell lines which may be due to variability in transfection efficiency or stochastic differences in expression within a population. Furthermore, between the different panels in (Fig. 2) we can observe that the fold change in fluorescence can range from a five-fold increase in fluorescence to a 350-fold increase. Some of these differences may be attributed to stochastic expression differences, fluorogen brightness, toxicity perceived by the cells or day-to-day variation in laser power. Therefore, we advise to always report fluorescence brightness as a relative readout compared to control conditions or pre-staining conditions. These issues might limit its applications in quantitative fluorescence microscopy.

Due to practical concerns, all the reversibility experiments were performed using a single biological repeat. However, in (Fig. S2), data from the three biological repeats with the fluorogens is depicted. From this data it can be appreciated that these behave similarly. Moreover, the experiments depicted in (Fig. 2) panels D, E, F and G were conducted using a different biological replicate compared to panels A, B and C. This further bolsters the fact that these trends, although they may not always be equally strong, occur independently of the specific isolate.

Even though we observe some variability and heterogeneity in staining, the strength of this tag is the capacity for live-cell imaging with multiple possible fluorogens. This is in stark contrast with HA labelling and antibody staining where cells have to be fixed in order to allow antibody targeting and imaging [30].

The viability of iFAST as a protein tag for live-cell imaging is demonstrated with the localisation of Erg11 and Ftr1, which are in accordance to the literature. Additional analysis also showed that for the Ftr1-iFAST strain, the tagging of the protein had no effect on the growth of the strain. However, the strain with an overexpression construct of Erg11-iFAST showed a reduction in growth rate in combination with a delay in the exponential phase compared to the parent strain (respectively *P*=0.0215, *P*<0.0001). From this we can conclude that there may be an adverse effect of tagging a protein-of-interest but that this effect is dependent on the manner in which it is attached, and the specific protein-of-interest and its transport throughout the cell [18,23, 24]. However, some background signal of Coral and Lime may be observed, this background is most obvious in (Fig. 4) where endogenous imaging of Ftr1 resulted in a significant background signal in the SC5314 parental strain treated with Lime. However, there is a continuous development of novel fluorogens for these fluorogen-activating proteins, which will be brighter and further increase the contrast [31].

The multi-colour characteristic of iFAST, which we also demonstrated by utilizing different fluorogens in the Erg11 and Ftr1 tagged strains, allows researchers to combine an iFAST-tagged protein with other tagged proteins or organic cell stainings without the requirement for the construction of new reporter constructs due to spectral overlaps. Even though significant spectral overlap was observed between Lime and Coral whilst using the most suitable excitation and emission filter settings for these fluorogens, theoretically Lime remains more suited for co-imaging with red or infra-red FPs or dyes whilst Coral is an excellent combination with blue FPs or dyes. Some of the spectral overlap was to be expected since the excitation and emission spectra of both Lime and Coral are relatively broad [6]. Furthermore, the versatility in absorption and emission of the fluorogens allows the system to be used for a wide range of excitation light and band filter combinations of different microscopes.

The reversible binding between iFAST and the fluorogens make it an ideal basis for the development of a real-time biosensor. This was reported with the original FAST protein in both a circularly permuted and split-FP setting and recently an improvement to this split-FP was discovered through an orthology-based screening [32,34]. Development of a similar system for *C. albicans* based on iFAST would greatly advance the research of protein-protein interactions in this species. Additionally, at least 60 versions of iFAST, such as mcFAST-L, mcFAST-Y, rFAST, gFAST, frFAST and even a smaller FAST, called nanoFAST, have been developed [28,29, 35, 36]. The combination palette of FAST and fluorogen is further expanded through the development of novel classes of fluorogen [31]. Due to practical concerns these were not pursued, but the iFAST reported here may be the starting point of further development of these fluorogen-activating proteins for the *Candida* research field.

In summary, this work describes and illustrates the application of a small fluorogenic tag with the ability to reversibly bind a fluorogen in *C. albicans*. We demonstrated the multi-colour facet of the protein tag resulting in green or red fluorescence, depending on the fluorogen. Furthermore, we highlighted the application of iFAST in live-cell imaging by tagging a membrane protein and the target of azole antifungal agents.

## supplementary material

10.1099/mic.0.001451 Uncited Supplementary Material 1.

## References

[1] Findley K, Oh J, Yang J, Conlan S, Deming C (2013). Topographic diversity of fungal and bacterial communities in human skin. Nature.

[2] Bougnoux M-E, Diogo D, François N, Sendid B, Veirmeire S (2006). Multilocus sequence typing reveals intrafamilial transmission and microevolutions of *Candida albicans* isolates from the human digestive tract. J Clin Microbiol.

[3] Beigi RH, Meyn LA, Moore DM, Krohn MA, Hillier SL (2004). Vaginal yeast colonization in nonpregnant women: a longitudinal study. Obstet Gynecol.

[4] Brown GD, Denning DW, Gow NAR, Levitz SM, Netea MG (2012). Hidden killers: human fungal infections. Sci Transl Med.

[5] Tebo AG, Pimenta FM, Zhang Y, Gautier A (2018). Improved chemical-genetic fluorescent markers for live cell microscopy. Biochemistry.

[6] Plamont M-A, Billon-Denis E, Maurin S, Gauron C, Pimenta FM (2016). Small fluorescence-activating and absorption-shifting tag for tunable protein imaging in vivo. Proc Natl Acad Sci U S A.

[7] Stagge F, Mitronova GY, Belov VN, Wurm CA, Jakobs S (2013). Snap-, Clip- and Halo-tag labelling of budding yeast cells. PLoS One.

[8] Salinas F, Rojas V, Delgado V, Agosin E, Larrondo LF (2017). Optogenetic switches for light-controlled gene expression in yeast. Appl Microbiol Biotechnol.

[9] Gomes AC, Miranda I, Silva RM, Moura GR, Thomas B (2007). A genetic code alteration generates a proteome of high diversity in the human pathogen *Candida albicans*. Genome Biol.

[10] Santos MAS, Tuite MF (1995). The CUG codon is decoded in vivo as serine and not leucine in *Candida albicans*. Nucleic Acids Res.

[11] Daniel E, Onwukwe GU, Wierenga RK, Quaggin SE, Vainio SJ (2015). ATGme: open-source web application for rare codon identification and custom DNA sequence optimization. BMC Bioinformatics.

[12] Puigbò P, Guzmán E, Romeu A, Garcia-Vallvé S (2007). OPTIMIZER: a web server for optimizing the codon usage of DNA sequences. Nucleic Acids Res.

[13] Van Genechten W, Demuyser L, Dedecker P, Van Dijck P (2020). Presenting a codon-optimized palette of fluorescent proteins for use in *Candida albicans*. Sci Rep.

[14] Fisher MC, Hawkins NJ, Sanglard D, Gurr SJ (1979). Worldwide emergence of resistance to antifungal drugs challenges human health and food security. Science.

[15] Tielker D, Eichhof I, Jaeger KE, Ernst JF (2009). Flavin mononucleotide-based fluorescent protein as an oxygen-independent reporter in *Candida albicans* and *Saccharomyces cerevisiae*. Eukaryot Cell.

[16] Shcherbakova DM, Verkhusha VV (2013). Near-infrared fluorescent proteins for multicolor in vivo imaging. Nat Methods.

[17] Smith EM, Gautier A, Puchner EM (2019). Single-molecule localization microscopy with the fluorescence-activating and absorption-shifting tag system. ACS Chem Biol.

[18] Ramanan N, Wang Y (1979). A high-affinity iron permease essential for *Candida albicans* virulence. Science.

[19] Subotić A, Swinnen E, Demuyser L, De Keersmaecker H, Mizuno H (2017). A bimolecular fluorescence complementation tool for identification of protein-protein interactions in *Candida albicans*. G3.

[20] Gietz RD, Schiestl RH, Willems AR, Woods RA (1995). Studies on the transformation of intact yeast cells by the LiAc/SS-DNA/PEG procedure. Yeast.

[21] Noble SM, Johnson AD (2005). Strains and strategies for large-scale gene deletion studies of the diploid human fungal pathogen *Candida albicans*. Eukaryot Cell.

[22] Rueden CT, Schindelin J, Hiner MC, DeZonia BE, Walter AE (2017). ImageJ2: imageJ for the next generation of scientific image data. BMC Bioinformatics.

[23] Kerstens W, Kremer A, Holtappels M, Borghgraef P, Lippens S (2020). Three-dimensional visualization of APEX2-tagged Erg11 in *Saccharomyces cerevisiae* using focused ion beam scanning electron microscopy. mSphere.

[24] Van Genechten W, Demuyser L, Duwé S, Vandenberg W, Van Dijck P (2021). Photochromic fluorophores enable imaging of lowly expressed proteins in the autofluorescent fungus *Candida albicans*. mSphere.

[25] Jain P, Sethi SC, Pratyusha VA, Garai P, Naqvi N (2018). Ras signaling activates glycosylphosphatidylinositol anchor biosynthesis via the GPI-*N*-acetylglucosaminyltransferase in *Candida albicans*. J Biol Chem.

[26] Pierce JV, Kumamoto CA (2012). Variation in *Candida albicans* EFG1 expression enables host-dependent changes in colonizing fungal populations. mBio.

[27] Hsu PC, Yang CY, Lan CY (2011). *Candida albicans* Hap43 is a repressor induced under low-iron conditions and is essential for iron-responsive transcriptional regulation and virulence. Eukaryot Cell.

[28] Li C, Tebo AG, Thauvin M, Plamont M-A, Volovitch M (2020). A far-red emitting fluorescent chemogenetic reporter for in vivo molecular imaging. Angew Chem Int Ed Engl.

[29] Tebo AG, Moeyaert B, Thauvin M, Carlon-Andres I, Böken D (2021). Orthogonal fluorescent chemogenetic reporters for multicolor imaging. Nat Chem Biol.

[30] Granger BL (2018). Accessibility and contribution to glucan masking of natural and genetically tagged versions of yeast wall protein 1 of *Candida albicans*. PLoS One.

[31] Myasnyanko IN, Gavrikov AS, Zaitseva SO, Smirnov AY, Zaitseva ER (2021). Color tuning of fluorogens for FAST fluorogen-activating protein. Chemistry.

[32] Tebo AG, Pimenta FM, Zoumpoulaki M, Kikuti C, Sirkia H (2018). Circularly permuted fluorogenic proteins for the design of modular biosensors. ACS Chem Biol.

[33] Tebo AG, Gautier A (2019). A split fluorescent reporter with rapid and reversible complementation. Nat Commun.

[34] Rakotoarison LM, Tebo AG, Böken D, Gautier A (2023). Improving split reporters of protein-protein interactions through orthology-based protein engineering. Cell Biol.

[35] Goncharuk MV, Baleeva NS, Nolde DE, Gavrikov AS, Mishin AV (2022). Structure-based rational design of an enhanced fluorogen-activating protein for fluorogens based on GFP chromophore. Commun Biol.

[36] Bottone S, Joliot O, Cakil ZV, El Hajji L, Rakotoarison L-M (2023). A fluorogenic chemically induced dimerization technology for controlling, imaging and sensing protein proximity. Nat Methods.

