# Supplementary material for: A multi-colour fluorogenic tag and its application in Candida albicans
Source: Microbiology (Reading). 2024 Mar 27;170(3):001451. doi: 10.1099/mic.0.001451 (PMC10995450; doi:10.1099/mic.0.001451)
Supplement: Uncited Supplementary Material 1. [file mic-170-01451-s001.pdf]

Supporting information for

A multi-colour fluorogenic tag and its application in Candida albicans

Jonas Devos[1], Patrick Van Dijck[1,*], Wouter Van Genechten[1]

1 Laboratory of Molecular Cell Biology, Institute of Botany and Microbiology, KU Leuven, 3001 Leuven, Belgium

* Patrick Van Dijck

Email: Patrick.vandijck@kuleuven.be

This PDF file includes:

Supplementary Figures 1 to 3

Supplementary Tables 1 to 4

Supplementary Information S1

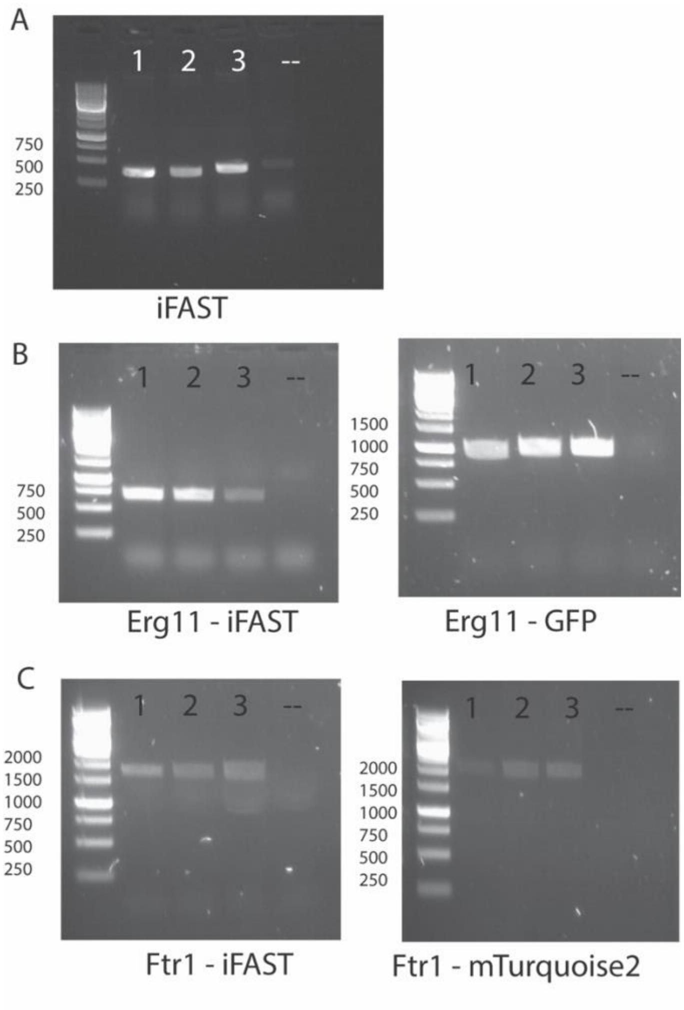

Supplementary figure 1: Assessment of correct construct integration using diagnostic PCR. PCR was performed utilizing Extaq polymerase on genomic DNA with primers listed in supplementary table 1. A) Diagnostic PCR on SN152 transformed with 2015-iFAST construct. Negative strain is the untransformed SN152 background (--). B) Diagnostic PCR on SC5314 transformed with a linear Erg11-iFAST construct (left) or a Erg11 – GFP construct (right). Diagnostic PCR was designed so that the forward primer anneals in Erg11 and the reverse primer anneals within the fluorescent tag. Untransformed SC5314 is the negative control (indicated with --) C) Diagnostic PCR on SC5314 transformed with a Ftr1 – iFAST (left) or Ftr1 – mTurquoise2 (right) construct. Diagnostic PCR was designed so that the forward primer anneals in Ftr1 and the reverse primer anneals within the fluorescent tag. Untransformed SC5314 is the negative control (indicated with --).

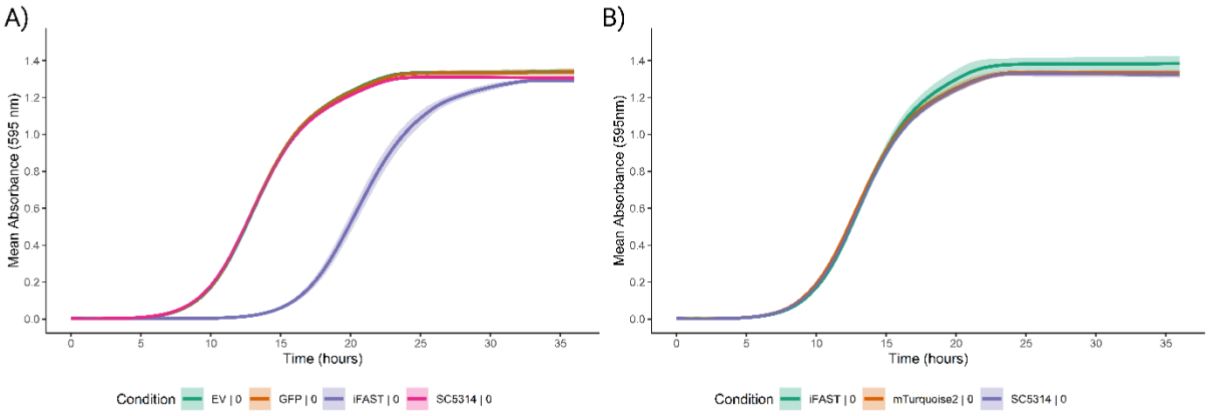

Supplementary figure 2: Assessment of iFAST tagging on growth of *C. albicans*. The data presented is the mean absorbance of the liquid cultures at 595 nm of two independent experiments with each three biological repeats and four technical repeats. A) Growth comparison of SC5315 containing an overexpression construct of Erg11-iFAST to the parental strain, an

empty vector containing strain, and SC5314 containing an overexpression construct of Erg11-GFP. B) Growth comparison of SC5314 with endogenously tagged Ftr1 with iFAST to the parental strain, and SC5314 with endogenously tagged Ftr1 using mTurquoise2. Data analysis was done on the slope of the exponential phase using one-way ANOVA and a Tukey post hoc test (Table S1)

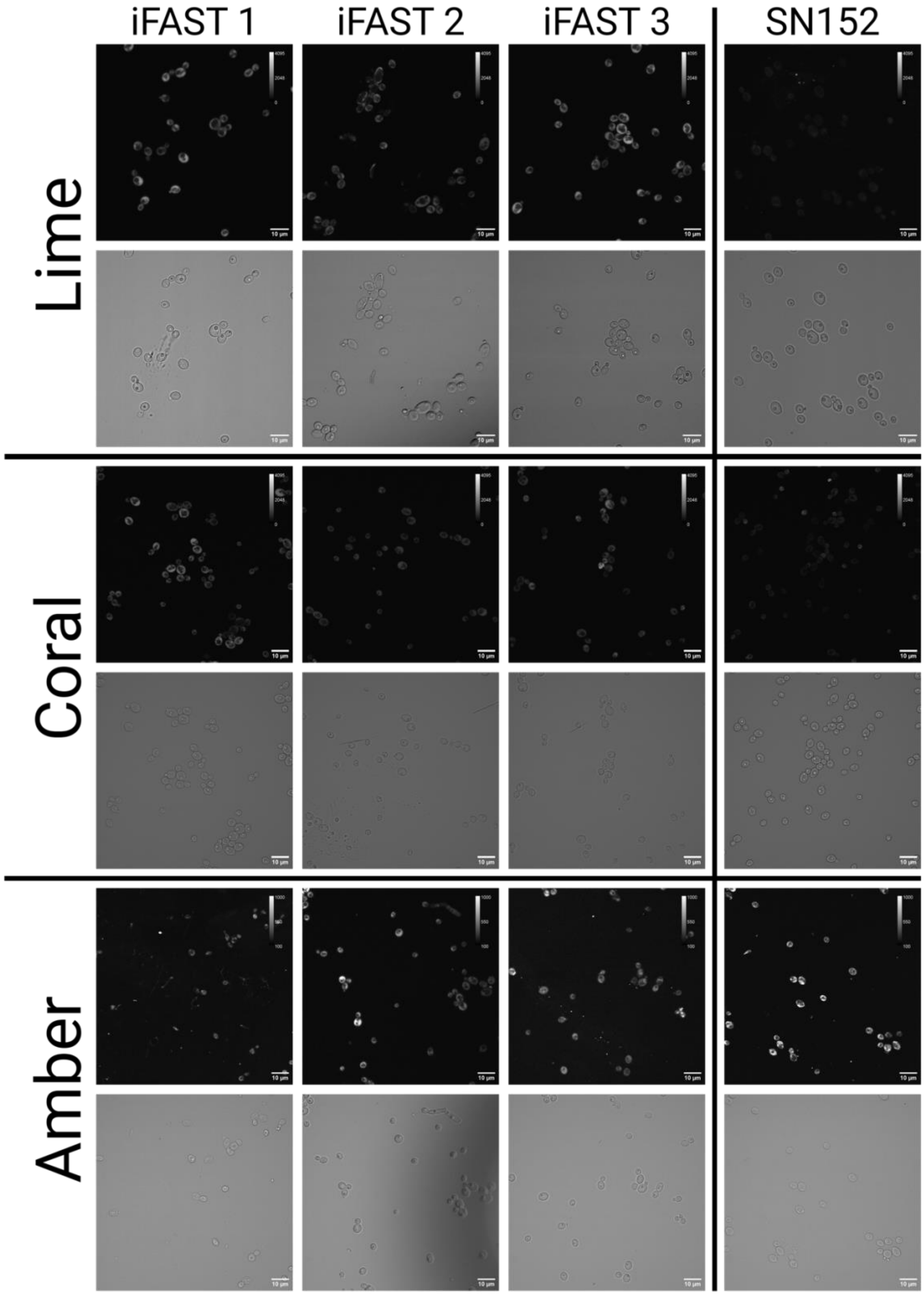

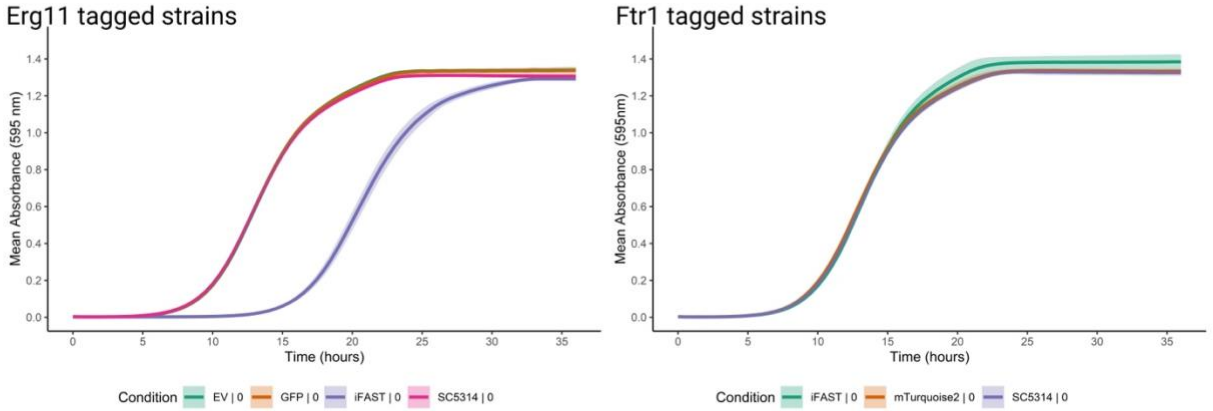

Supplementary figure 3: Imaging of three biological repeats of iFAST expressing strains. SN152 and the iFAST-integrated strain were grown overnight at 30°C in medium lacking methionine and cysteine and diluted to an OD_{600} of 0.2 before growing to mid-exponential phase. Images are taken with identical settings to those of figure 2.

Supplementary table 1: List of the primers used for the construction of the cytosolic iFAST expression plasmids and for the endogenous tagging of C. albicans genes with iFAST using PCR-fragment assisted integration.

Name	Sequence
Construction of plasmids for cytoplasmatic expression of iFAST	
**2015N-iFAST Fw**	AATACCCTCCCCAGAAAACTATGGAACATGTTGCCTTTG
**2015N-iFAST Rv**	CACCAGAACCTCCACCTCCGTTAAACCCTTTTGACAAACAC
Construction of the pFA6 plasmids	
**pFA6-iFAST Fw**	CTGAAGCTTCGTACGCTGCAATGGAACATGTTGCCTTTG
**pFA6-iFAST Rv**	CATGCATTTACTTATAATGGTTAAACCCTTTTGACAAACAC
Primers used for endogenous tagging of genes in *C. albicans*	
***FTR1*-iFAST Fw**	AAGTTGATGAAACTTCATCAAACAAATTGATCGAATCCAAAGAAAACAAAGGAGGTGGAGGTTCTGGTGGAGGTGGTTCAATGGAACATGTTGCCTTTGG
***SAT1-FTR1* Rv**	CACAGTCTCTTGCCTTATTCTTTTAGTTGTTGAATAATAATTAACTAAGTTTAGGC<br>GTCATCCTGTGCTC
FTR1 – mT2	GGATGAATTGTTCACTAAACTTCAACAACAAAACTTTGGTCAAGCTGCAGAAGTTGATGAAACTTCATCAAACAAATTGATCGAATCCAAAGAAAACAAAGGAGGTGGAGGTTCTGGTGGAGGTGGTTCAATGGTTTCTAAAGGTGAAGA
Primers used for the construction of the ERG11-iFAST/GFP overexpression plasmid	
***ERG11* (iFAST) Fw**	TAATCATTCAAAATGCTGCACATGGCTATTGTTGAAACTG
***ERG11* (iFAST) Rv**	AAACATACAAGTTTCTCTTTTTTC
**iFAST Fw**	AAAGAGAAACTTGTATGTTTGGAGGTGGAGGTTCTGGTGGAGGTGGTTCAATGGAACATGTTGCCTTTG
**iFAST Rv**	CGACGGTATCGATGACGTCGTTAAACCCTTTTGACAAACAC
**ERG11 FW (GFP)**	taatcattcaaaatgctgcacATGGCTATTGTTGAAACTG
**ERG11 RV (GFP)**	tgaaccacctccaccagaacctccacctcctgaaccacctccaccagaacctccacctccAAACATACAAGTTTCTCTTTTTTC
**GFP FW**	ggaggtggaggttctggtggaggtggttcaggaggtggaggttctggtggaggtggttcaATGTCTAAAGGTGAAGAATTATTC

**GFP RV**	gatttccagaatttcactccTTATTTGTACAATTCATCCATAC
Primers used for diagnostic PCR's	
iFAST FW	ATGGAACATGTTGCCTTTG
iFAST RV	TTAAACCCTTTTGACAAACAC
Erg11 FW	GCTGCTGCCAAAGCTAATTC
Erg11 – iFAST RV	cgacggtatcgatgacgtcgTTAAACCCTTTTGACAAACAC
Erg11 – GFP RV	CCGCTCGAGTTATTTGTACAATTCATCCA
Ftr1 FW	GATTTTGGTGAATCTCCCTCC
Ftr1 – iFAST RV	cgacggtatcgatgacgtcgTTAAACCCTTTTGACAAACAC
Ftr1 – mT2 RV	tagaaagtataggaacttccTTATTTGTACAATTCATCCATAC

Supplementary table 2: Overview of all plasmids that were used and constructed during this work.

Plasmid	Description	Source
**2015N**	*MET3*p *CmLEU2*	Subotić *et al.*, 2015
**CIp10**	CIp10 plasmid where URA3 marker was replaced with *NAT1* gene	Murad *et al.,* 2000
**pFA6a-yEmVenus**	yEmVenus *ACT1*p-*SAT1*	This study
**2015N-iFAST**	Same as 2015N but containing iFAST under control of the *MET3*p	This study, Addgene #209415
**pFA6a-iFAST**	Same as pFA6a but with the yEmVenus sequence replaced by the iFAST sequence	This study, Addgene #209414
**ERG11-iFAST**	Same as CIp10 but containing ERG11-iFAST under control of the *ACT1*p	This study

Supplementary table 3: List with all used strains during this work.

Strain	Genotype	Source
**SC5314**	Wild type	Noble *et al.,* 2005
**SN152**	*arg4Δ/arg4Δ leu2Δ/leu2Δ his1Δ/his1Δ URA3/ura3Δ*::imm[434] *IRO1/iro1Δ*::imm[434]	Subotić *et al.,* 2015
**SN152-2015N**	SN152 with integration of the empty 2015N plasmid	This study
**SN152-Venus**	SN152 with integration of the 2015N-Venus plasmid	This study
**iFAST**	SN152 with integration of the 2015N-iFAST plasmid	This study
**Ftr1-iFAST**	SC5314 with endogenous tagging of *FTR1* with iFAST	This study
**Ftr1-mTurquoise2**	SC5314 with endogenous tagging of *FTR1* with mTurquoise2	This study

**Erg11-iFAST**	SC5314 but with integration of the ErgRG11-iFAST plasmid	This study
**Erg11-GFP**	SC5314 with integration of the Erg11-GFP plasmid	This study
**SC5314-CIp10**	C5314 with integration of the empty CIp10 plamsid	This study

Supplementary table 4: Statistical analysis of the log phase of the growth curve data

To statistically compare the growth curves, the slope of the exponential phase and the lag phase of each biological repeat of each strain/ condition was calculated using the QurvE package (v1.1) . The strains/conditions were compared using one-way ANOVA with a Tukey post hoc test.

Table 4.1: Statistical analysis on the iFAST integrated strain with Lime

ANOVA

	Growth rate				Lag phase			
	Sum Sq	Df	F Value	Pr(>F)	Sum Sq	Df	F Value	Pr(>F)
Sample	0.0938	3	10.3985	0.0039	3.3658	3	1.0584	0.4189
Residuals	0.0241	8	NA	NA	8.4805	8	NA	NA

Tukey Post Hoc

	Growth rate	
	diff	p.adj
iFAST | 10-iFAST | 0	-0.0208	0.9647
iFAST | 20-iFAST | 0	0.0335	0.8747
iFAST | 50-iFAST | 0	-0.195	0.0104
iFAST | 20-iFAST | 10	0.0544	0.6358
iFAST | 50-iFAST | 10	-0.1741	0.0193
iFAST | 50-iFAST | 20	-0.2285	0.0041

Table 4.2: Statistical analysis on the iFAST integrated strain with Coral

ANOVA

	Growth rate				Lag phase			
	Sum Sq	Df	F Value	Pr(>F)	Sum Sq	Df	F Value	Pr(>F)
Sample	0.1054	3	92.3236	0	8.6588	3	27.2395	1e-04
Residuals	0.003	8	NA	NA	0.8477	8	NA	NA

Tukey Post Hoc

	Growth rate		Lag phase	
	diff	p.adj	diff	p.adj
iFAST | 10-iFAST | 0	9e-04	0.9999	1.0974	0.014
iFAST | 20-iFAST | 0	-0.0149	0.7861	1.1378	0.0115
iFAST | 50-iFAST | 0	-0.2206	0	2.3995	1e-04
iFAST | 20-iFAST | 10	-0.0158	0.7573	0.0404	0.9986
iFAST | 50-iFAST | 10	-0.2215	0	1.302	0.0052
iFAST | 50-iFAST | 20	-0.2057	0	1.2616	0.0063

Table 4.3: Statistical analysis on the iFAST integrated strain with Amber

ANOVA

	Growth rate				Lag phase			
	Sum Sq	Df	F Value	Pr(>F)	Sum Sq	Df	F Value	Pr(>F)
Sample	0.053	3	8.4893	0.0072	3.6202	3	3.9878	0.0523
Residuals	0.0166	8	NA	NA	2.4209	8	NA	NA

Tukey Post Hoc

	Growth rate	
	diff	p.adj
iFAST | 10-iFAST | 0	0.1048	0.0866
iFAST | 20-iFAST | 0	0.115	0.0587
iFAST | 50-iFAST | 0	-0.0396	0.7192
iFAST | 20-iFAST | 10	0.0102	0.9922
iFAST | 50-iFAST | 10	-0.1444	0.0196
iFAST | 50-iFAST | 20	-0.1546	0.0136

Table 4.4: Statistical analysis on effect of iFAST integration

ANOVA

	Growth rate				Lag phase			
	Sum Sq	Df	F Value	Pr(>F)	Sum Sq	Df	F Value	Pr(>F)
Sample	0.0022	3	4.3868	0.042	21.3837	3	69.4529	0
Residuals	0.0013	8	NA	NA	0.821	8	NA	NA

Tukey Post Hoc

	Growth rate		Lag phase	
	diff	p.adj	diff	p.adj
iFAST | 0-EV | 0	0.0321	0.0609	-2.5605	0
SN152 | 0-EV | 0	0.0088	0.8332	1.1036	0.0124
Venus | 0-EV | 0	0.0288	0.0947	-0.1997	0.8686
SN152 | 0-iFAST | 0	-0.0233	0.1973	3.6641	0
Venus | 0-iFAST | 0	-0.0033	0.9886	2.3608	1e-04
Venus | 0-SN152 | 0	0.02	0.2971	-1.3033	0.0047

Table 4.5: Statistical analysis on effect of tagging Erg11 with iFAST.

ANOVA

	Growth rate				Lag phase			
	Sum Sq	Df	F Value	Pr(>F)	Sum Sq	Df	F Value	Pr(>F)
Sample	0.0126	3	11.0947	0.0032	93.2256	3	133.6084	0
Residuals	0.003	8	NA	NA	1.8607	8	NA	NA

Tukey Post Hoc

	Growth rate		Lag phase	
	diff	p.adj	diff	p.adj
GFP | 0-EV | 0	-0.0078	0.9589	0.3285	0.837
iFAST | 0-EV | 0	-0.0825	0.0037	6.4366	0
SC5314 | 0-EV | 0	-0.022	0.5426	-0.273	0.8969
iFAST | 0-GFP | 0	-0.0747	0.0067	6.1081	0
SC5314 | 0-GFP | 0	-0.0142	0.8099	-0.6015	0.466
SC5314 | 0-iFAST | 0	0.0606	0.0215	-6.7096	0

Table 4.6: Statistical analysis on effect of tagging Ftr1 with iFAST

ANOVA

	Growth rate				Lag phase			
	Sum Sq	Df	F Value	Pr(>F)	Sum Sq	Df	F Value	Pr(>F)
Sample	2e-04	2	1.2691	0.347	0.2061	2	4.0633	0.0766
Residuals	5e-04	6	NA	NA	0.1522	6	NA	NA

Supplementary information 1: Full genetic and protein sequence for the *Candida albicans* optimized iFAST

Gene encoding *C. albicans* optimized iFAST:

ATGGAACATGTTGCCTTTGGTAGCGAAGATATCGAGAATACTCTAGCGAAAATGGATGATG
GCCAATTAGATGGATTAGCCTTTGGTGCTATACAGCTTGATGGTGATGGCAATATTTTACA
GTATAATGCAGCTGAAGGAGACATAACAGGGAGAGATCCCAAACAAGTGATTGGCAAGA
ACTTCTTCAAAGACGTAGCACCAGGTACAGATTCTCCTGAATTTTACGGGAAATTCAAGG
AAGGAGTAGCATCAGGTAACTTGAATACCATGTTCGAGTGGATGATTCCGACGAGTAGGG
GGCCAACCAAGGTTAAAATTCACATGAAGAAGGCTTTGAGTGGTGACTCCTATTGGGTGT
TTGTCAAAAGGGTTTAA

Protein sequence of *C. albicans* optimized iFAST:

MEHVAFGSEDIENTLAKMDDGQLDGLAFGAIQLDGDGNILQYNAAEGDITGRDPKQVIGKNF
FKDVAPGTDSPEFYGKFKEGVASGNLNTMFEWMIPTSRGPTKVKIHMKKALSGDSYWVFVK
RV
